# Clinical characteristics and overall survival prognostic nomogram for invasive cribriform carcinoma of breast: a SEER population-based analysis

**DOI:** 10.1186/s12885-021-07895-5

**Published:** 2021-02-16

**Authors:** Jiameng Liu, Xiaobin Zheng, Zhonghua Han, Shunguo Lin, Hui Han, Chunsen Xu

**Affiliations:** 1 Department of Breast Surgery, Fujian Medical University Union Hospital, Fuzhou, 350001 Fujian Province China; 2 Department of General Surgery, Fujian Medical University Union Hospital, Fuzhou, 350001 Fujian Province China; 3 Breast Cancer Institute, Fujian Medical University, Fuzhou, 350001 Fujian Province China; 4 Department of Radiotherapy, Fujian Medical University Cancer Hospital, Fuzhou, 350000 Fujian Province China

**Keywords:** Invasive cribriform carcinoma, Overall survival, Prognosis, SEER, Nomogram

## Abstract

**Background:**

The prognositc factors in patient with invasive cribriform carcinoma (ICC) of breast is still remain controversal. The study aims to establish a nomogram to predict the survival outcomes in patients with ICC based on the Surveillance, Epidemiology and End Results (SEER) database.

**Methods:**

We retrieved SEER database for clinical data about patients including ICC and infiltrating ductal carcinoma (IDC) from 2004 to 2015. Kaplan-Meier survival was used to compare the difference survival outcomes between ICC and IDC. ICC patients were randomly allocated to training cohort and validation cohort. A nomogram was built to predict individual patient’s 3-year and 5-year survival status for ICC. The established TMN model and the newly established nomogram was further evaluated by the concordance index (C-index) and the decision curve analysis (DCA).

**Results:**

Comparing the baseline clinical data between IDC and ICC, a significant of smaller tumor mass, less infiltrated lymph nodes, lower metastases rate, better tumor differentiation degree, higher proportion of estrogen receptor (ER) and progesterone receptor (PR) positive and lower rate of chemotherapy and radiotherapy was found in ICC. Age at diagnosis, marriage status, tumor location, T stage, M stage, ER status, surgery were independent significant prognostic factors for the overall survival (OS). A significantly higher C-index was found in nomogram compared with established TNM model in validation cohort.

**Conclusions:**

The prognosis of ICC patients is better than that of IDC patients. The nomogram is recommended for future patient with ICC to survival analysis.

**Supplementary Information:**

The online version contains supplementary material available at 10.1186/s12885-021-07895-5.

## Background

Breast cancer has the highly mortality rate in female worldwide. Breast cancer includes many pathological subtypes, among them ICC is a rare but unique one, characterized by mild to moderate cytological atypia nest surround by a dense fibrous stroma, with an incidence rate of less than 4% [1]. Distinct from breast cancer, ICC is considered to have higher survival rate, so in clinical practice, its uniqueness should be considered [2]. However, some limitations exist in previous reports including small number cohort or evident bias result from limited follow-up period due to the fact that ICC patients are relatively rare [3–5]. Most of ICC’s local and system best controlled treatment methods are inferred from IDC’s treatment experience, and have not been strictly verified in ICC patients. The TNM is the most wildly used staging system. It indicate the objective tumor load and metastasis status but have limited capacity to characterize the biological behavior features and guide decision making [6]. Because ICC lacks a unique prognostic evaluation system, ICC treatment is relatively uniform. Nomogram is confirmed as an reliable and alternative prognosis assessment tool in many cancer types [7–9] and even thought to be a emerging new standard [10]. In this study, we aim to build a reliable and high accuracy nomogram to predict individual ICC patient’s survival outcome based on clinical and pathological data from SEER database.

## Materials & methods

### Data source and study population

The Surveillance, Epidemiology, and End Results (SEER) database aims to collect information about cancer characteristics, cancer incidence and results. We acquired permission to download and analyze data for academic purpose (reference number: 14737-Nov2018). This study does not contain any experiments on humans as well as animals and/or the use of human tissue samples performed by any of the authors. The inclusion and exclusion criteria for extracting and screening data from SEER database including people from 18 regions (1973–2016), released on August 8th, 2019 were as follows. Inclusion criteria: (1) the diagnosing year ranged from 2004 to 2015, (2) the primary site of tumor was breast, and (3) histological types were confined to 8500/3 (IDC) and 8201/3 (ICC) according to ICD-0-3. Exclusion criteria: (1) patients with unknown information of race, diagnosing year, marital status or important clinicopathological data, (2) patients younger than 20 years old, (3) patients with a history of other cancer, (4) patients with less than 1 month survival after diagnosis, and (5) patient’s diagnoses were only depend on biopsy or autopsy. Patients with ICC that met criteria were randomly allocated to training cohort (*n* = 532) and validation cohort (*n* = 228).

### Endpoint and statistical analysis

Overall survival (OS) was defined as the time from the date of diagnosis to the date of death due to any cause or the last followup. Race was divided into white, black and other races; estrogen receptor (ER) is divided into positive and negative; progesterone receptor (PR) is divided into positive and negative; human epidermal growth factor receptor 2 (HER-2) was divided into negative, positive and unknown; the age grouping is implemented through the X-tile Software (Fig. S1). Age is divided into < 68,68 ~ 78,> = 79; and marital status was reclassified as married, single (never married or with a domestic partner) or divorced (separated, divorced and widowed). The clinicopathological features of different groups were analyzed by chi-square test or Fisher exact test. The survival curve was generated by the Kaplan-Meier method. The log-rank test was used to assess the difference in survival of each group. The three-year and five-year overall survival were calculated by the life table method. In the training cohort, the Cox regression model, hazard ratios (HRs) and 95% confidence intervals (CIs) were used for variable analysis to adjust prognostic variables. Variables selected by univariate Cox regression with *p*-value < 0.05 were selected for multivariate analysis using forword stepwise regression. In multivariate analysis, T, N, and M variables were used instead of stage variables to avoid multicollinearity. According to the results of the multivariate Cox regression hazards model, the nomogram model was constructed using the rms package in R software. The nomogram model was verified by the identification and calibration measurements of the training cohort and validation cohort. C-index, which measures the difference in predictive power between observation and prediction, was used to evaluate the discriminative power of the nomogram model [11]. The receiver operating characteristic (ROC) curve was used to verify the nomogram model. The use of marginal estimates to establish a calibration map of the model represents the calibration between the predicted survival rate and the observed survival rate of the nomogram model. Evaluation of the clinical effectiveness and benefit of the prediction model by decision curve analysis (DCA) [12]. C-index and DCA were used to compare the nomogram model with the AJCC 6th TNM staging system in the validation cohort.

Analyses *p* values were two-sided, and values of < 0.05 were considered statistically significant. Statistical analysis was performed with IBM SPSS Statistics 20 and R software (version 3.6.3).

## Results

### Clinical and pathological characteristics

We included a total of 305,644 eligible patients in this study. The median age of 304,884 (99.75%) IDC patients was 59 years old, and the median age of 760 (0.25%) ICC patients was 61 years old. Among patients over 67 years old, the proportion of ICC patients was higher than that of IDC patients (*p* < 0.001). The proportion of male patients with ICC is larger (*p* < 0.05). ICC patients had a greater proportion of breast cancer on the left (*p* < 0.05). In addition, ICC patients had a lower T stage (76.10% vs. 60.80%, *p* < 0.001), lower lymph node involvement rate (83.20% vs. 66.10%, *p* < 0.001), and less distant metastasis (98.70% vs. 96.30%, *p* < 0.05), and well tumor differentiation degree (59.57% vs. 17.40%, *p* < 0.001) than IDC patients. ICC had a higher rates of ER-positive (95.40% vs. 78.50%, *p* < 0.001), a higher rate of PR-positive status (89.50% vs. 68.30%, *p* < 0.001), and a lower rate of HER-2 positive (2.90% vs. 9.30%, *p* < 0.001). ICC patients received less radiotherapy and chemotherapy. There was no statistical difference between the proportion of ICC and IDC receiving surgery (97.20% vs. 95.90%, *p* = 0.07) (Table 1). In the training cohort and verification cohort of ICC patients, except for the HER-2 status (*p* = 0.04), the remaining 16 variables had no statistically significant difference between the two groups (Supplementary Table 1).

**Table 1:** The characteristics of 305,644 breast cancer patients

Characteristics	ICC,N(%)	IDC,N(%)	N(%)	*P*-value
	760 (0.2%)	304,884 (99.8%)	305,644 (100%)	
Age				< 0.001
< 68	499 (65.7%)	221,196 (72.6%)	221,695 (72.5%)	
68–78	162 (21.3%)	55,204 (18.1%)	55,366 (18.1%)	
79+	99 (13.0%)	28,484 (9.3%)	28,583 (9.4%)	
Race				0.331
White	601 (79.1%)	241,929 (79.4%)	242,530 (79.4%)	
Black	76 (10.0%)	33,747 (11.1%)	33,823 (11.1%)	
Other	83 (10.9%)	29,208 (9.6%)	29,291 (9.6%)	
Sex				0.002
Female	747 (98.3%)	302,644 (99.3%)	303,391 (99.3%)	
Male	13 (1.7%)	2240 (0.7%)	2253 (0.7%)	
Marital				0.082
Married	421 (55.4%)	180,589 (59.2%)	181,010 (59.2%)	
Single	121 (15.9%)	46,315 (15.2%)	46,436 (15.2%)	
Divorced	218 (28.7%)	77,980 (25.6%)	78,198 (25.6%)	
Site				0.200
Other	299 (39.3%)	119,554 (39.2%)	119,853 (39.2%)	
502	112 (14.7%)	37,497 (12.3%)	37,609 (12.3%)	
503	41 (5.4%)	17,801 (5.8%)	17,842 (5.8%)	
504	262 (34.5%)	107,370 (35.2%)	107,632 (35.2%)	
505	46 (6.1%)	22,662 (7.4%)	22,708 (7.4%)	
Laterality				0.033
Left	415 (54.6%)	154,712 (50.7%)	155,127 (50.8%)	
Right	345 (45.4%)	150,172 (49.3%)	150,517 (49.2%)	
Grade				< 0.001
I + II	705 (92.8%)	184,512 (60.5%)	185,217 (60.6%)	
II + IV	55 (7.2%)	120,372 (39.5%)	120,427 (39.4%)	
AJCC stage				< 0.001
I	513 (67.5%)	148,311 (48.6%)	148,824 (48.7%)	
II	208 (27.4%)	110,720 (36.3%)	110,928 (36.3%)	
III	29 (3.8%)	34,605 (11.4%)	34,634 (11.3%)	
IV	10 (1.3%)	11,248 (3.7%)	11,258 (3.7%)	
T stage				< 0.001
T1	578 (76.1%)	185,375 (60.8%)	185,953 (60.8%)	
T2	152 (20.0%)	93,007 (30.5%)	93,159 (30.5%)	
T3	20 (2.6%)	14,938 (4.9%)	14,958 (4.9%)	
T4	10 (1.3%)	11,564 (3.8%)	11,574 (3.8%)	
N stage				< 0.001
N0	632 (83.2%)	201,664 (66.1%)	202,296 (66.2%)	
N1	103 (13.6%)	74,583 (24.5%)	74,686 (24.4%)	
N2	18 (2.4%)	18,517 (6.1%)	18,535 (6.1%)	
N3	7 (0.9%)	10,120 (3.3%)	10,127 (3.3%)	
M stage				0.001
M0	750 (98.7%)	293,636 (96.3%)	294,386 (96.3%)	
M1	10 (1.3%)	11,248 (3.7%)	11,258 (3.7%)	
ER status				< 0.001
Negative	35 (4.6%)	65,425 (21.5%)	65,460 (21.4%)	
Positive	725 (95.4%)	239,459 (78.5%)	240,184 (78.6%)	
PR status				< 0.001
Negative	80 (10.5%)	96,508 (31.7%)	96,588 (31.6%)	
Positive	680 (89.5%)	208,376 (68.3%)	209,056 (68.4%)	
HER-2 status				< 0.001
Negative	360 (47.4%)	134,383 (44.1%)	134,743 (44.1%)	
Positive	22 (2.9%)	28,250 (9.3%)	28,272 (9.2%)	
Unknown	378 (49.7%)	142,251 (46.7%)	142,629 (46.7%)	
Surgery				0.070
No	21 (2.8%)	12,381 (4.1%)	12,402 (4.1%)	
Yes	739 (97.2%)	292,503 (95.9%)	293,242 (95.9%)	
Radiaotherapy				0.032
No	363 (47.8%)	133,868 (43.9%)	134,231 (43.9%)	
Yes	397 (52.2%)	171,016 (56.1%)	171,413 (56.1%)	
Chemotherapy				< 0.001
No	592 (77.9%)	165,041 (54.1%)	165,633 (54.2%)	
Yes	168 (22.1%)	139,843 (45.9%)	140,011 (45.8%)	

### Survival analysis

**Fig. 1:**
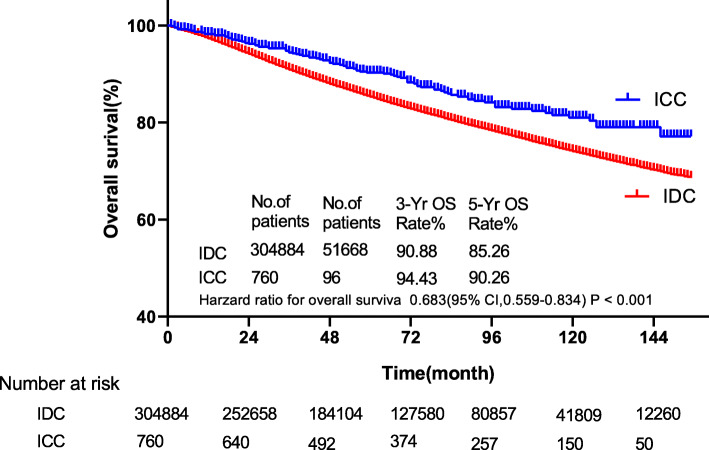
The survival of patients with ICC and IDC by Kaplan-Meier analysis. Patients with ICC had better survival (HR = 0.683, 95% CI, 0.559–0.834,p < 0.001) with 3- and 5-year OS rates of 94.43 and 90.26% vs. 90.88 and 85.26% in IDC patients, respectively

The median follow-up time was 60 months (1–155 months). The survival of ICC patients was significantly prolonged than IDC patients by KM analysis (*p* < 0.001). The 3-year and 5-year OS rates of ICC were 94.43 and 90.26%, respectively. While the 3-year and 5-year OS rates of IDC patients were 90.88 and 85.26%, respectively (Fig. 1). Through univariate Cox regression analysis, the histological type of ICC was a better prognostic factor for breast cancer (HR = 0.683; 95% CI, 0.559–0.834 *p* < 0.001).

### Prognostic factors in ICC

**Fig. 2:**
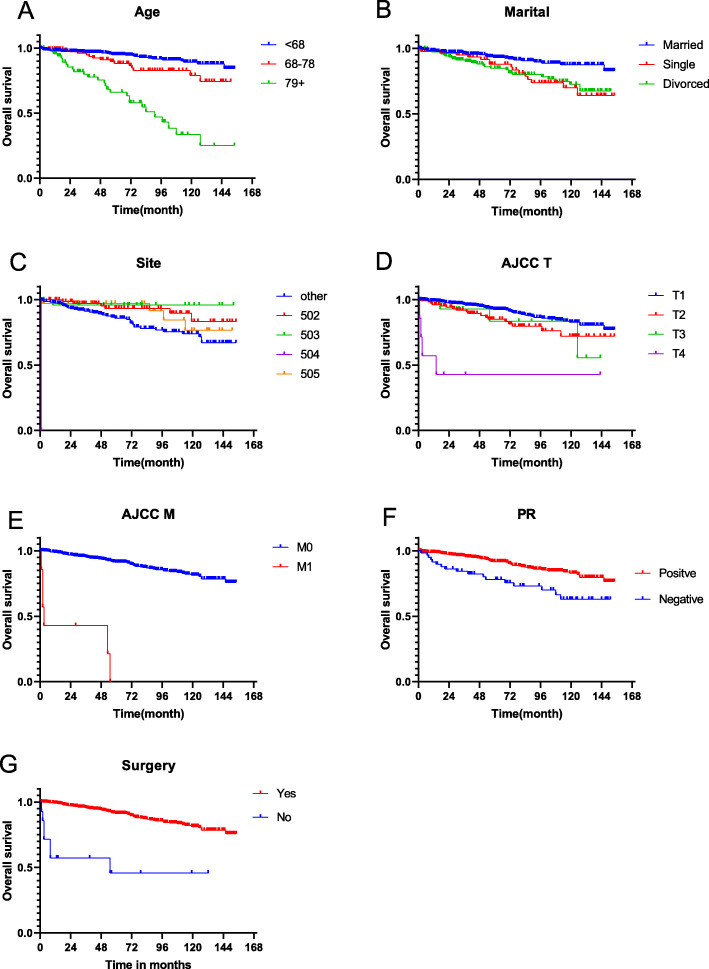
Kaplan-Meier OS curves for patients with ICC according to different independent prognostic factors. **a**-**g** Kaplan-Meier OS curves for patients with ICC according to **a** age, **b** marital, **c** site, **d** AJCC T, **e** AJCC M, **f** PR and **g** surgery

The Cox regression model was used in the training cohort to discover the factors affecting the prognosis in ICC. The univariate analysis showed that the age, marital status, tumor location, stage, T, M, PR, surgery, radiotherapy and chemotherapy had statistically significant differences in survival prognostic factors, while not the N stage (*p* = 0.070) and ER (*p* = 0.749). Further multivariate Cox analysis showed that age, marital status, tumor location, T, M, PR, and surgery were independent prognostic factors (Table 2). Kaplan-Meier survival curves of each independent prognostic factor was show in Fig. 2.

**Table 2:** Univariate and multivariate Cox regression analysis of Overall survival (ICC Training Cohort)

Characteristics	Univariate analysis		Multivariate analysis	
	HR(95%CI)	*P*-value	HR(95%CI)	*P*-value
Age		< 0.001		< 0.001
< 68	Reference		Reference	
68–78	2.025 (1.071–3.827)	0.030	2.576 (1.262–5.258)	0.009
79+	8.326 (4.848–14.299)	< 0.001	14.362 (7.309–28.222)	< 0.001
Race		0.228		
White	Reference			
Black	1.508 (0.746–3.051)	0.253		
Other	0.546 (0.198–1.506)	0.242		
Sex		0.355		
Female	Reference			
Male	1.944 (0.476–7.945)	0.355		
Marital		0.003		< 0.001
Married	Reference		Reference	
Single	2.343 (1.251–4.389)	0.008	4.087 (2.019–8.275)	< 0.001
Divorced	2.371 (1.382–4.068)	0.002	1.66 (0.888–3.105)	0.113
Site		0.038		0.022
Other	Reference		Reference	
502	0.392 (0.165–0.929)	0.033	0.485 (0.201–1.171)	0.108
503	0.206 (0.028–1.504)	0.119	0.217 (0.025–1.87)	0.164
504	0.525 (0.307–0.897)	0.018	0.45 (0.25–0.813)	0.008
505	0.55 (0.196–1.545)	0.257	0.309 (0.102–0.941)	0.039
Laterality		0.317		
Left	Reference			
Right	0.782 (0.484–1.266)	0.317		
Grade				
I + II	Reference			
II + IV	1.057 (0.425–2.628)	0.904		
AJCC stage		< 0.001		
I	Reference			
II	1.413 (0.829–2.409)	0.204		
III	1.901 (0.678–5.335)	0.222		
IV	32.616 (13.295–80.013)	< 0.001		
T stage		< 0.001		< 0.001
T1	Reference		Reference	
T2	1.777 (1.026–3.079)	0.040	1.656 (0.887–3.094)	0.113
T3	1.711 (0.531–5.516)	0.368	2.175 (0.651–7.266)	0.207
T4	12.661 (4.522–35.453)	0.000	40.392 (9.908–164.669)	< 0.001
N stage		0.070		
N0	Reference			
N1	1.034 (0.527–2.029)	0.922		
N2	0.768 (0.106–5.55)	0.793		
N3	4.78 (1.49–15.333)	0.009		
M stage		< 0.001		< 0.001
M0	Reference		Reference	
M1	28.362 (11.839–67.949)	< 0.001	52.29 (13.76–198.705)	< 0.001
ER status		0.749		
Negative	Reference			
Positive	1.208 (0.379–3.847)	0.749		
PR status		0.001		0.001
Negative	Reference		Reference	
Positive	0.408 (0.236–0.706)	0.001	0.363 (0.199–0.662)	0.001
HER-2 status		0.199		
Negative	Reference			
Positive	2.273 (0.677–7.629)	0.184		
Unknown	0.781 (0.436–1.398)	0.405		
Surgery		< 0.001		0.019
No	Reference		Reference	
Yes	0.127 (0.058–0.278)	< 0.001	0.27 (0.09–0.809)	0.019
Radiaotherapy		< 0.001		
No	Reference			
Yes	0.343 (0.205–0.574)	< 0.001		
Chemotherapy		0.009		
No	Reference			
Yes	0.353 (0.161–0.771)	0.009		

*Abbreviations*: *ICC* Invasive cribriform carcinoma, *IDC* Infiltrating ductal carcinoma, *502* Upper-inner quadrant of breast, *503* Lower-inner quadrant of breast, *504* Upper-outer quadrant of breast, *505* Lower-outer quadrant of breast, *ER* Estrogen receptor, *PR* Progesterone receptor, *HER-2* Human epidermal growth factor receptor 2

### Construction and validation of nomogram

**Fig. 3:**
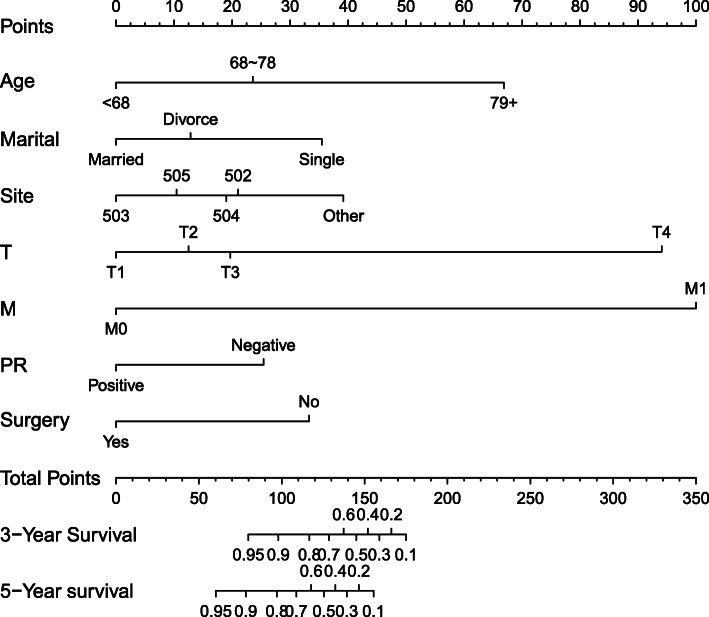
Nomogram predicted 3- and 5-year overall survival for patients with ICC. The nomogram is used by summing the points identified on the top scale for each independent covariate. The total points projected to the bottom scale indicated the % probability of the 3- and 5-year OS

The independent prognostic factors identified by the Cox regression (age, marital status, tumor location, T, M, PR, and whether it is surgically treated) were used for building a nomogram model to predict the OS in ICC (Fig. 3). The nomogram model showed that M stage had the greatest impact on prognosis, and the smallest is PR. All subtypes of all variables are assigned scores (Table 3).

**Table 3:** Point assignment and prognostic score in the nomogram (ICC Training Cohort)

Variable	Score
Age	
< 68	0
68–78	24
79+	67
Marital	
Married	0
Single	35
Divorced	13
Site	
Other	39
502	21
503	0
504	19
505	10
T stage	
T1	0
T2	12
T3	20
T4	94
M stage	
M0	0
M1	100
PR status	
Negative	25
Positive	0
Surgery	
No	33
Yes	0

**Fig. 4:**
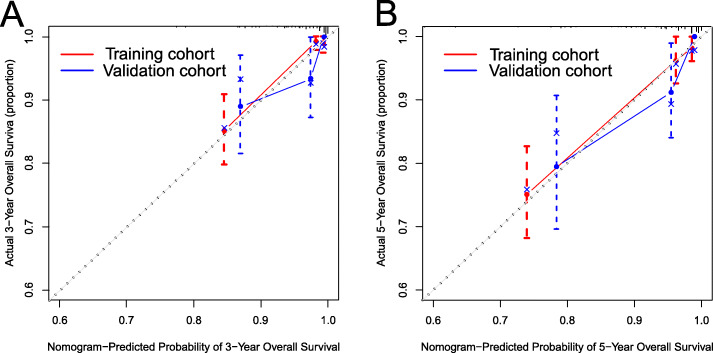
The calibration plot for predicting 3- and 5-year overall survival for patients with ICC. Calibration plot of nomogram prediction of **a** 3-year and **b** 5-year OS of patients with ICC

**Fig. 5:**
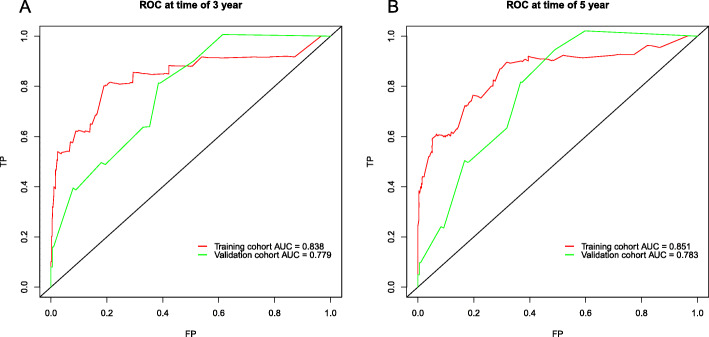
Discriminatory accuracy for predicting OS assessed by ROC analysis calculating AUC. There-year OS in the training and validation cohort (**a**). Five-year OS in the training and validation cohort (**b**)

The nomogram model was internally and externally verified. On the one hand, the internal verification in the training cohort, presented that the C-index predicted by OS was 0.845 (95% CI [0.788–0.902]). On the other hand, the externally verified shows that the C-index predicted from the validation cohort by the OS was 0.807 (95% CI [0.728–0.876]). The calibration plots showed the good consistency between the nomogram prediction and the actual observation in training cohort and validation cohort (Fig. 4). The ROC of the training and verification cohort is shown in the Fig. 5.

**Fig. 6:**
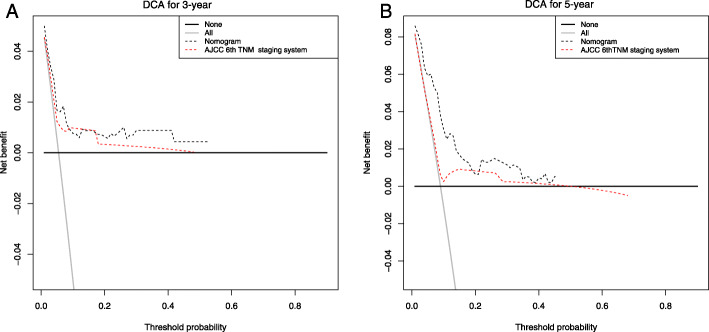
DCA for the Nomogram and AJCC 6th TNM staging system in the validation cohort. DCA in prediction of patients at 3-year (**a**) and 5-year (**b**)

The C-index of the OS predicted by the nomogram in the verification cohort was 0.807 (95% CI, 0.728–0.876), that was even higher than the AJCC 6th TNM staging system (C-index = 0.591; 95% CI [0.505–0.677]). The DCA was used to contrast the availability and benefits of the nomogram and the AJCC 6th TNM staging system. Compared with the AJCC 6th TNM staging system, the 3-year and 5-year DCA curves of the nomogram showed a bigger net benefit across a series of death risks in the validation cohort (Fig. 6).

## Discussion

ICC of breast cancer is a rare histological type with a low degree of malignancy. A series of previous studies showed that ICC has a good prognosis and has characteristics different from other histological breast cancer types [13, 14]. Also in our study, the prognosis of ICC patients was significantly better than that of IDC patients. However, ICC is difficult to distinguish from other types of breast cancer in imaging [15].

The prognosis of ICC patients are still controversial. The effectiveness of breast cancer subtypes as prognostic factors has been widely accepted by clinicians at present. Currently NCCN and ASCO guidelines recommend the use of ER and PR status as the significant prognostic factors in medical decision-making. However, in this study, multivariate Cox analysis indicated that ER was not an independently prognostic factor for ICC. Part of the reason is that the positive rate of ER in our study is too high, which will make it difficult for us to determine the prognostic effect of ER status.

In this study, whether surgical treatment is an independent prognostic factor for ICC, but whether chemotherapy treatment is not an independent prognostic factor that affects the prognosis of ICC. Zhang et al. also believe that due to the good prognosis of ICC, chemotherapy is not required [3]. Chemotherapy in this study is not an independent prognostic factor, which may be caused by the late stage of patients requiring chemotherapy. Therefore, whether ICC patients need chemotherapy needs further study in the future.

The prognosis of traditional TNM staging system evaluation patients only includes T stage, N stage, and M stage, which does not include other biological factors. From the nomogram we constructed, we can see that in addition to T stage and M stages, the age of diagnosis, marital status, tumor location, PR status also has a greater impact on prognosis. But, the N stage in our study had no statistical significance for the prognosis in the Cox regression analysis. ICC can be divided into simple type and mixed type. The mixed type has higher lymph node positive rate and poor prognosis [3, 5, 13]. However, our research cannot distinguish the subtypes of ICC. Therefore, we speculate that N stage may be less important than T and M staging in Cox regression analysis or that N stage is not a prognostic factor due to the bias generated by the N stage that cannot distinguish the ICC subtypes in our research.

A published study analyzed the prognosis of ICC using the SEER data. The results showed that ICC patients were more likely to be elderly women with smaller tumors, better tumor differentiation degree, fewer lymph node metastases, and higher ER and PR positive rates. The prognosis is better than IDC patients [16]. As far as we know, this is the first study to build a nomogram in ICC based on a large sample. This nomogram has better accuracy (in the training cohort C-index 0.845) and clinical usability than the TNM staging system.

Limitations of this study include that we failed to distinguish ICC subtypes. ICC is divided into simple and mixed types, which have different prognostic results. Second, SEER data lacks information about Ki-67, chemotherapy regimens, endocrine therapy, and vascular invasion. In addition, our study did not verify the nomogram with multiple centers. Finally, the study is a retrospective cohort study, not a prospective cohort study. But, our study had a new understanding of the clinicopathological characteristics and prognosis of patients with ICC.

## Conclusions

ICC patients have smaller tumors, less lymph node invasion, less distant metastasis rate, higher frequency of well tumor differentiation degree, higher ER positive and PR positive rates, and less chemotherapy and radiotherapy. The prognosis of ICC patients is significantly better than that of IDC patients. Our research is the first to build a ICC nomogram model.

## Supplementary Information

**Additional file 1: Supplementary Table 1.** The characteristics of 760 patients of ICC. **Fig. S1.** The optimal cut-off value for age.

## Data Availability

These data were publicly available for use in accordance with a limited use agreement for SEER research data: Surveillance, Epidemiology, and End Results (SEER) Program (https://seer.cancer.gov) SEER*Stat Database.

## Abbreviations

ICC: Invasive cribriform carcinoma
IDC: Infiltrating ductal carcinoma
502: Upper-inner quadrant of breast
503: Lower-inner quadrant of breast
504: Upper-outer quadrant of breast
505: Lower-outer quadrant of breast
ER: Estrogen receptor
PR: Progesterone receptor
HER-2: Human epidermal growth factor receptor 2
