# Supplementary material for: Clinical characteristics and overall survival prognostic nomogram for invasive cribriform carcinoma of breast: a SEER population-based analysis
Source: BMC Cancer. 2021 Feb 16;21:168. doi: 10.1186/s12885-021-07895-5 (PMC7887783; doi:10.1186/s12885-021-07895-5)
Supplement: Supplementary file 1 — Additional file 1: Supplementary Table 1. The characteristics of 760 patients of ICC. Fig. S1. The optimal cut-off value for age. [file 12885_2021_7895_MOESM1_ESM.docx]

**Clinical characteristics and overall survival prognostic nomogram for invasive cribriform carcinoma of breast: a SEER population-based analysis**

Jiameng Liu^1,3,4†^, Xiaobin Zheng^2†^, Zhonghua Han^1,3,4^, Shunguo Lin^1,3,4^, Hui Han^1,3,4^, Chunsen Xu^1,3,4*^

^1^ Department of Breast Surgery, Fujian Medical University Union Hospital, Fuzhou, Fujian Province, 350001, China

^2^ Department of Radiotherapy，Fujian Medical University Cancer Hospital, Fuzhou, Fujian Province, 350000,China

^3^ Department of General Surgery, Fujian Medical University Union Hospital, Fuzhou, Fujian Province, 350001, China

^4^ Breast Cancer Institute, Fujian Medical University, Fuzhou, Fujian Province, 350001, China

^†^Jiameng Liu and Xiaobin Zheng have contributed equally to the work.

^†^Jiameng Liu and Xiaobin Zheng are co-frst authors.

*Corresponding Author:

Chunsen Xu^1,3,4^

NO.29, Xinquan Road, Fuzhou, Fujian, 350001, China

Email address: [xuchunsen@yeah.net](mailto:xuchunsen@yeah.net)

Phone:+86-13306900948

Supplementary Table 1. The characteristics of 760 patients of ICC.

| Characteristics | Training cohort,N(%) | Validation cohort,N(%) | N(%) | P-value |
| --- | --- | --- | --- | --- |
|  | 532（70.0%） | 228（30.0%） | 760（100%） |  |
| Age |  |  |  | 0.826 |
| <68 | 353（66.4%） | 146（64.0%） | 499（65.7%） |  |
| 68-78 | 111（20.9%） | 51（22.4%） | 162（21.3%） |  |
| 79+ | 68（12.8%） | 31（13.6%） | 99（13.0%） |  |
| Race |  |  |  | 0.651 |
| White | 425（79.9%） | 176（77.2%） | 601（79.1%） |  |
| Black | 50（9.4%） | 26（11.4%） | 76（10.0%） |  |
| Other | 57（10.7%） | 26（11.4%） | 83（10.9%） |  |
| Sex |  |  |  | 0.764 |
| Female | 522（98.1%） | 225（98.7%） | 747（98.3%） |  |
| Male | 10（1.9%） | 3（1.3%） | 13（1.7%） |  |
| Marital |  |  |  | 0.393 |
| Married | 302（56.8%） | 119（52.2%） | 421（55.4%） |  |
| Single | 85（16.0%） | 36（15.8%） | 121（15.9%） |  |
| Divorced | 145（27.3%） | 73（32.0%） | 218（28.7%） |  |
| Site |  |  |  | 0.212 |
| Other | 200（37.6%） | 99（43.4%） | 299（39.3%） |  |
| 502 | 78（14.7%） | 34（14.9%） | 112（14.7%） |  |
| 503 | 25（4.7%） | 16（7.0%） | 41（5.4%） |  |
| 504 | 194（36.5%） | 68（29.8%） | 262（34.5%） |  |
| 505 | 35（6.6%） | 11（4.8%） | 46（6.1%） |  |
| Laterality |  |  |  | 0.691 |
| Left | 288（54.1%） | 127（55.7%） | 415（54.6%） |  |
| Right | 244（45.9%） | 101（44.3%） | 345（45.4%） |  |
| Grade |  |  |  | 0.445 |
| I+II | 496（93.2%） | 209（91.7%） | 705（92.8%） |  |
| II+IV | 36（6.8%） | 19（8.3%） | 55（7.2%） |  |
| AJCC stage |  |  |  | 0.986 |
| I | 360（67.7%） | 153（67.1%） | 513（67.5%） |  |
| II | 144（27.1%） | 64（28.1%） | 208（27.4%） |  |
| III | 21（3.9%） | 8（3.5%） | 29（3.8%） |  |
| IV | 7（1.3%） | 3（1.3%） | 10（1.3%） |  |
| T stage |  |  |  | 0.97 |
| T1 | 404（75.9%） | 174（76.3%） | 578（76.1%） |  |
| T2 | 106（19.9%） | 46（20.2%） | 152（20.0%） |  |
| T3 | 15（2.8%） | 5（2.2%） | 20（2.6%） |  |
| T4 | 7（1.3%） | 3（1.3%） | 10（1.3%） |  |
| N stage |  |  |  | 0.867 |
| N0 | 443（83.3%） | 189（82.9%） | 632（83.2%） |  |
| N1 | 73（13.7%） | 30（13.2%） | 103（13.6%） |  |
| N2 | 11（2.1%） | 7（3.1%） | 18（2.4%） |  |
| N3 | 5（0.9%） | 2（0.9%） | 7（0.9%） |  |
| M stage |  |  |  | 1 |
| M0 | 525（98.7%） | 225（98.7%） | 750（98.7%） |  |
| M1 | 7（1.3%） | 3（1.3%） | 10（1.3%） |  |
| ER status |  |  |  | 0.851 |
| Negative | 24（4.5%） | 11（4.8%） | 35（4.6%） |  |
| Positive | 508（95.5%） | 217（95.2%） | 725（95.4%） |  |
| PR status |  |  |  | 0.606 |
| Negative | 58（10.9%） | 22（9.6%） | 80（10.5%） |  |
| Positive | 474（89.1%） | 206（90.4%） | 680（89.5%） |  |
| HER-2 status |  |  |  | 0.040 |
| Negative | 262（49.2%） | 98（43.0%） | 360（47.4%） |  |
| Positive | 19（3.6%） | 3（1.3%） | 22（2.9%） |  |
| Unknown | 251（47.2%） | 127（55.7%） | 378（49.7%） |  |
| Surgery |  |  |  | 0.81 |
| no | 14（2.6%） | 7（3.1%） | 21（2.8%） |  |
| yes | 518（97.4%） | 221（96.9%） | 739（97.2%） |  |
| Radiaotherapy |  |  |  | 0.384 |
| no | 260（48.90%） | 103（45.2%） | 363（47.8%） |  |
| yes | 272（51.10%） | 125（54.8%） | 397（52.2%） |  |
| Chemotherapy |  |  |  | 0.295 |
| no | 420（78.9%） | 172（75.4%） | 592（77.9%） |  |
| yes | 112（21.1%） | 56（24.6%） | 168（22.1%） |  |

Abbreviations:ICC,invasive cribriform carcinoma; IDC, infiltrating ductal carcinoma; 502=upper-inner quadrant of breast; 503=lower-inner quadrant of breast; 504=upper-outer quadrant of breast; 505=lower-outer quadrant of breast; ER, estrogen receptor; PR, progesterone receptor; HER-2, human epidermal growth factor receptor 2.

Fig.S1. The optimal cut-off value for age.


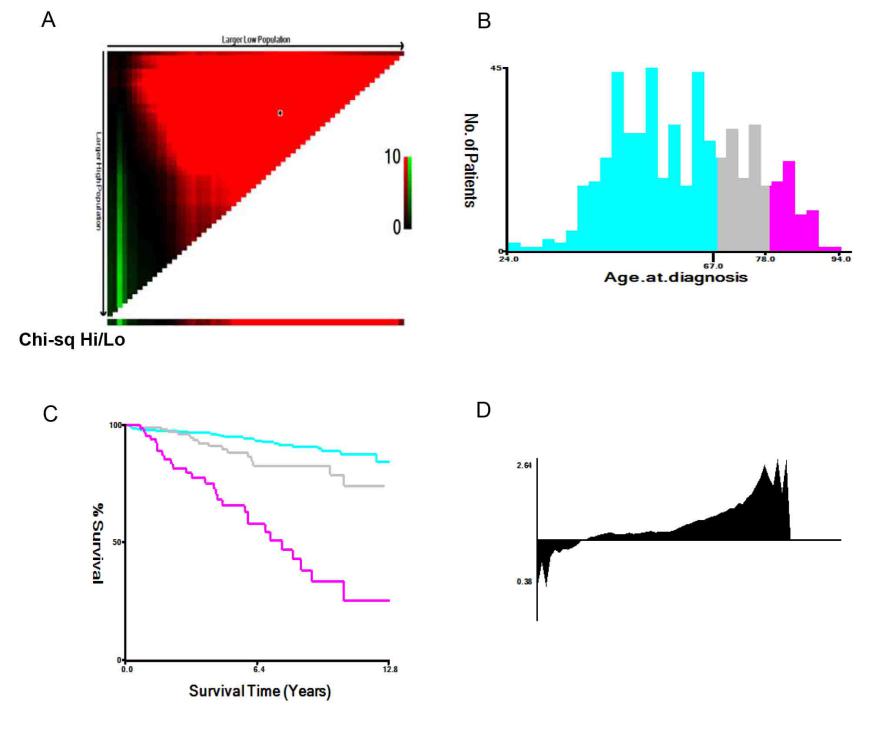


(A) The optimal cut-off value highlighted by the black circle in the rectangular X-tile plot.(low risk group, age<68; medium risk group, age 68-78 and high risk group, age 79+.) (B) The histogram of the entire cohort. (C) The Kaplan-Meier plot: The OS curve of young, older and oldest patients. The age of 68 and 78 is chosen as the optimal cut-off value. (D) The relative risks (RRs) for all cut-off values from low to high (left to right, x-axis). The RRs are calculated as: events in the older group / event risk in the younger group.
